# 2-Methyl-4-(4-nitro­phen­yl)but-3-yn-2-ol: crystal structure, Hirshfeld surface analysis and computational chemistry study

**DOI:** 10.1107/S2056989019010284

**Published:** 2019-07-23

**Authors:** Ignez Caracelli, Julio Zukerman-Schpector, Ricardo S. Schwab, Everton M. da Silva, Mukesh M. Jotani, Edward R. T. Tiekink

**Affiliations:** a Departamento de Física, Universidade Federal de São Carlos, 13565-905 São Carlos, SP, Brazil; b Departamento de Química, Universidade Federal de São Carlos, 13565-905 São Carlos, SP, Brazil; c Department of Physics, Bhavan’s Sheth R. A. College of Science, Ahmedabad, Gujarat 380001, India; d Research Centre for Crystalline Materials, School of Science and Technology, Sunway University, 47500 Bandar Sunway, Selangor Darul Ehsan, Malaysia

**Keywords:** crystal structure, acetyl­ene, hydrogen bonding, Hirshfeld surface analysis, NCI plots, computational chemistry

## Abstract

The di-substituted acetyl­ene residue in the title compound, C_11_H_11_NO_3_, is capped at either end by di-methyl­hydroxy and 4-nitro­benzene groups; the nitro substituent is close to co-planar with the ring to which it is attached [dihedral angle = 9.4 (3)°]. The most prominent feature of the mol­ecular packing is the formation, *via* hy­droxy-O—H⋯O(hy­droxy) hydrogen bonds, of hexa­meric clusters about a site of symmetry . The aggregates are sustained by 12-membered {⋯OH}_6_ synthons and have the shape of a flattened chair. The clusters are connected into a three-dimensional architecture by benzene-C—H⋯O(nitro) inter­actions, involving both nitro-O atoms. The aforementioned inter­actions are readily identified in the calculated Hirshfeld surface. Computational chemistry indicates there is a significant energy, primarily electrostatic in nature, associated with the hy­droxy-O—H⋯O(hy­droxy) hydrogen bonds. Dispersion forces are more important in the other identified but, weaker inter­molecular contacts.

## Abstract 2

In the title mol­ecule, di-methyl­hydroxy and 4-nitro­benzene groups cap a central di-substituted acetyl­ene residue. The extended structure features flattened, hexa­meric clusters sustained by hy­droxy-O—H⋯O(hy­droxy) hydrogen bonds.

## Chemical context   

Protected acetyl­enes represent a highly privileged class of synthetic inter­mediates for the construction of a variety of different organic compounds (Tan *et al.*, 2013 ▸). The preparation of protected aryl­acetyl­enes can be achieved by the palladium-catalysed Sonogashira cross-coupling of mono-protected acetyl­enes, such as tri­methyl­silyl­acetyl­ene (TMSA), triisopropysilyl­acetyl­ene (TIPSA) and 2-methyl-3-butyn-2-ol (MEBYNOL), with aryl halides (Hundertmark *et al.*, 2000 ▸; Erdélyi & Gogoll, 2001 ▸). Despite the relevance of protected acetyl­enes, the release of the protecting group remains a challenge. While tri­alkyl­silyl groups can be readily removed by treatment with bases or fluoride salts under mild reaction conditions, tri­alkyl­silyl­acetyl­enes are rather expensive, in comparison to MEYBNOL, thereby limiting their use to small-scale synthesis. Thus, MEBYNOL can be viewed as one alternative to other acetyl­ene sources. Nevertheless, the reaction conditions for the release of the 2-hy­droxy­isopropyl protecting group usually requires harsh reaction conditions. Hence, several synthetic routes combine the release of the terminal acetyl­ene with a further transformation, without the isolation of the inter­mediate (Li *et al.*, 2015 ▸). It was in the context of such considerations that the title acetyl­ene compound, (I), previously reported (Bleicher *et al.*, 1998 ▸), was isolated and crystallized. Herein, the crystal and mol­ecular structures of (I) are described along with a detailed analysis of the mol­ecular packing by Hirshfeld surface analysis, non-covalent inter­action plots and computational chemistry.
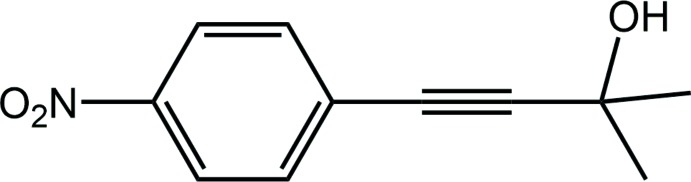


## Structural commentary   

**Figure 1:**
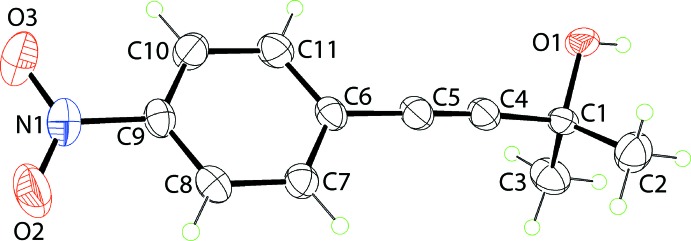
The mol­ecular structure of (I), showing the atom-labelling scheme and displacement ellipsoids at the 35% probability level.

The mol­ecular structure of (I), Fig. 1 ▸, features a di-substituted acetyl­ene residue. At one end, the acetyl­ene terminates with a di-methyl­hydroxy substituent and at the other end, with a 4-nitro­benzene group. The nitro group is slightly inclined out of the plane of the benzene ring to which it is connected, with the dihedral angle between the planes being 9.4 (3)°.

## Supra­molecular features   

**Figure 2:**
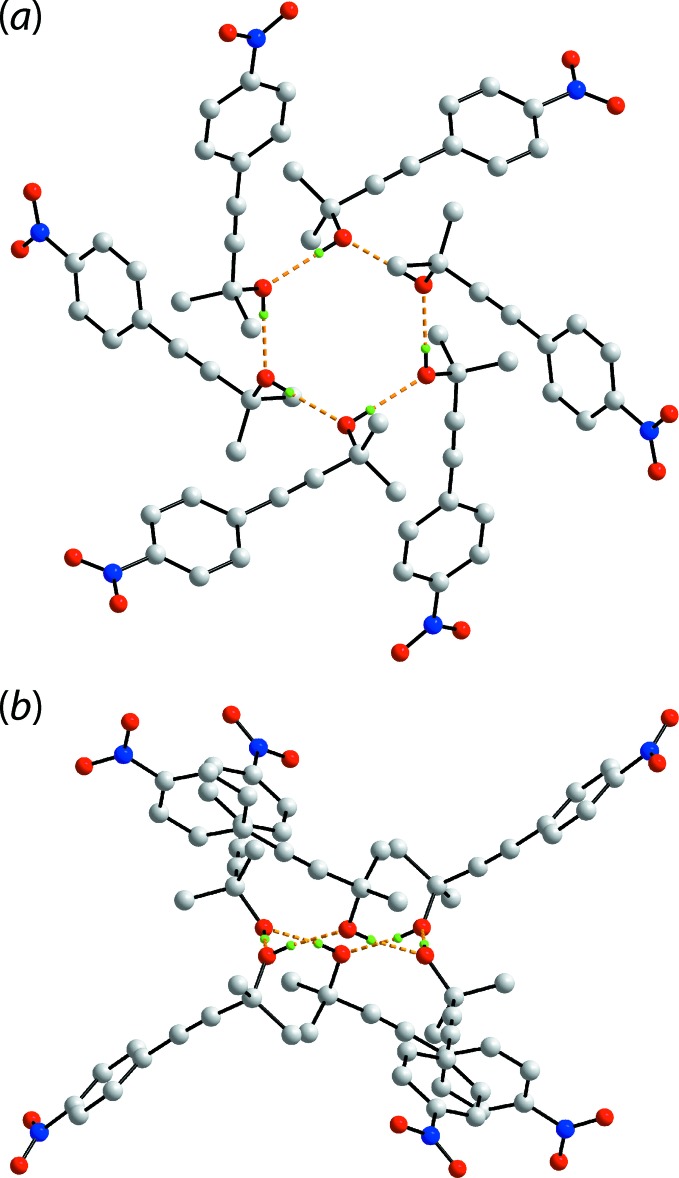
Hydrogen bonding in the crystal of (I): (*a*) an end-on view of the hexa­gon sustained by hy­droxy-O—H⋯O(hy­droxy) hydrogen bonding (shown as orange dashed lines) and (*b*) a side-on view. Non-participating hydrogen atoms have been removed for reasons of clarity.

**Figure 3:**
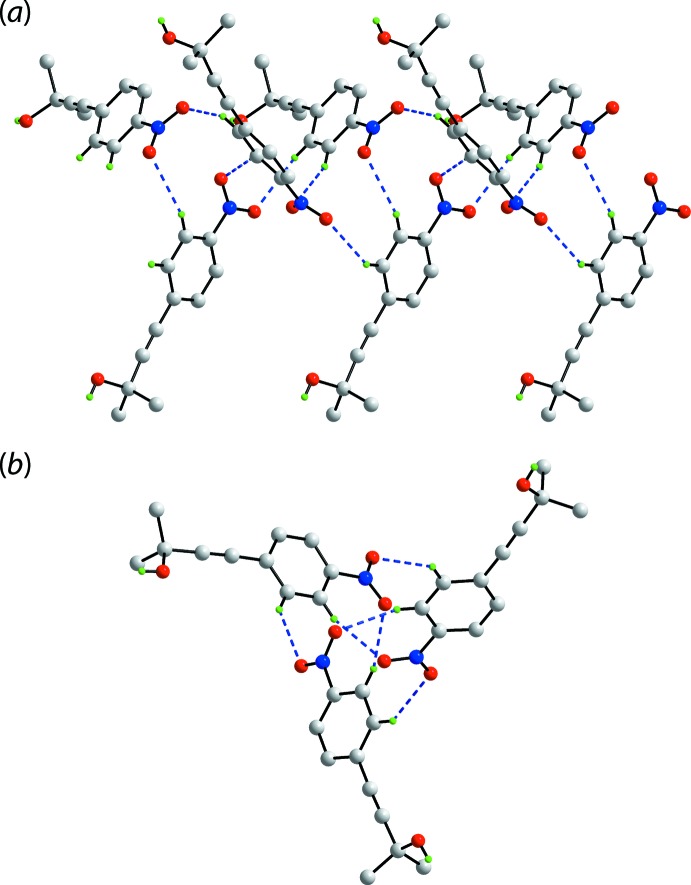
Details of benzene-C—H⋯O(nitro) inter­actions (shown as blue dashed lines) in the crystal of (I): (*a*) a view of the supra­molecular chain along the *c*-axis direction and (*b*) an end-on view of the chain.

**Figure 4:**
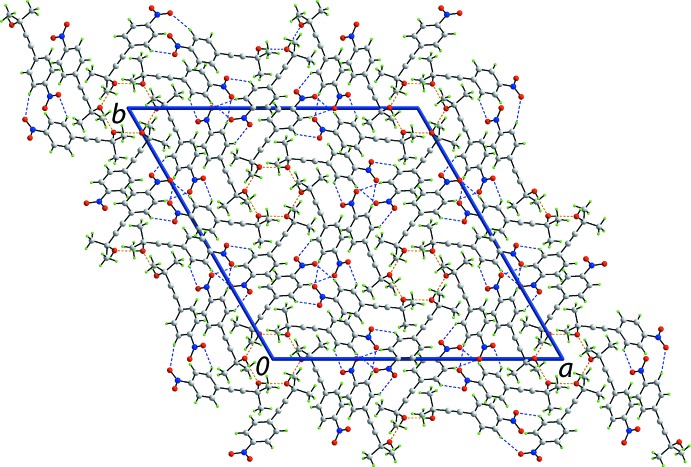
A view of the unit-cell contents of (I) shown in projection down the *c* axis. The hy­droxy-O—H⋯O(hy­droxy) hydrogen bonding and benzene-C—H⋯O(nitro) inter­actions are shown as orange and blue dashed lines, respectively.

The spectacular feature of the mol­ecular packing of (I) is the presence of hexa­meric clusters connected by hy­droxy-O—H⋯O(hy­droxy) hydrogen bonds, Table 1 ▸. As seen from Fig. 2 ▸(*a*), the six-mol­ecule aggregates are sustained by 12-membered {⋯OH}_6_ synthons. The aggregates are disposed about a site of symmetry 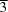 so the rings have the shape of a flattened chair, Fig. 2 ▸(*b*). The crystal also features weak benzene-C—H⋯O(nitro) inter­actions, involving both nitro-O atoms. In essence, one nitro group of one mol­ecule forms two such inter­actions with two symmetry-related mol­ecules to form a supra­molecular chain along the *c*-axis direction with helical symmetry (3_1_ screw axis), Fig. 3 ▸(*a*). An end-on view of the chain is shown in Fig. 3 ▸(*b*). These weak benzene-C—H⋯O(nitro) inter­actions serve to link the six-mol­ecule aggregates into a three-dimensional architecture, Fig. 4 ▸.

## Hirshfeld surface analysis   

**Figure 5:**
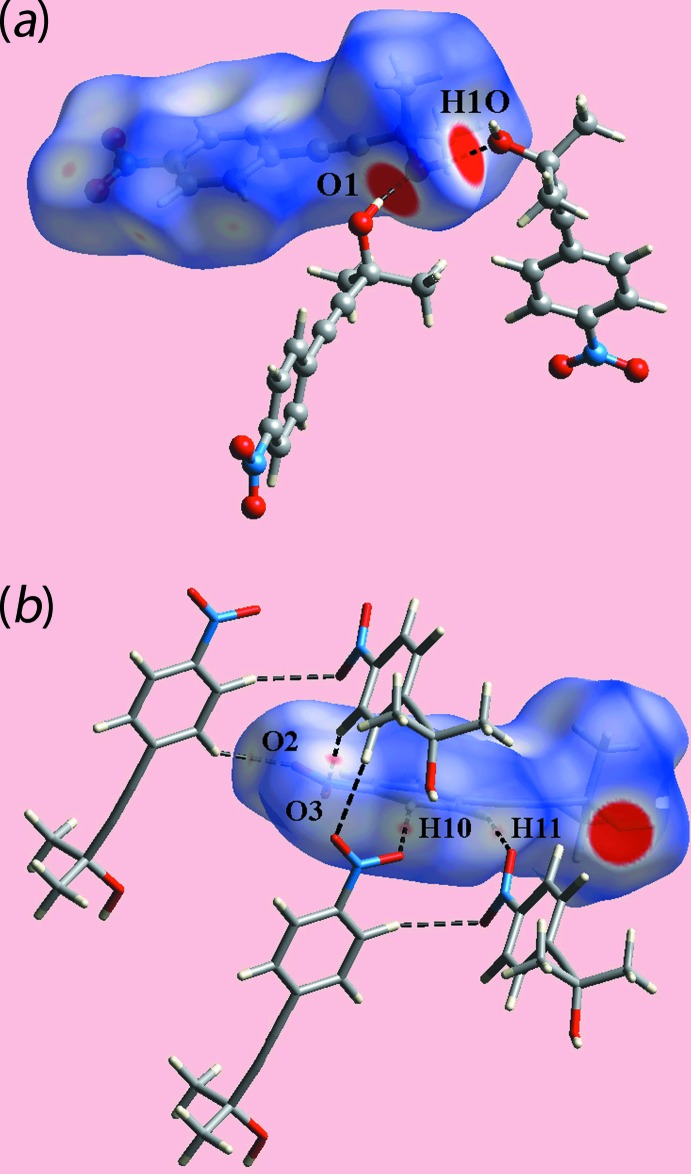
Two views of the Hirshfeld surface for (I) mapped over *d*_norm_: (*a*) in the range −0.202 to +1.400 arbitrary units and (*b*) in the range −0.102 to +1.400 arbitrary units, highlighting, respectively, inter­molecular O—H⋯O and C—H⋯O inter­actions through black dashed lines.

**Figure 6:**
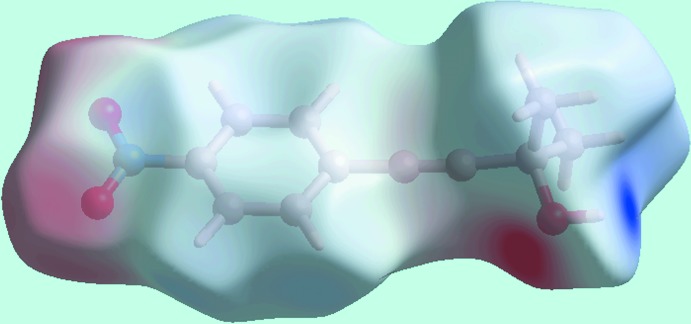
A view of the Hirshfeld surface for (I) mapped over the electrostatic potential in the range −0.098 to + 0.180 atomic units. The red and blue regions represent negative and positive electrostatic potentials, respectively, and show the acceptors and donors of inter­molecular inter­actions, respectively.

**Figure 7:**
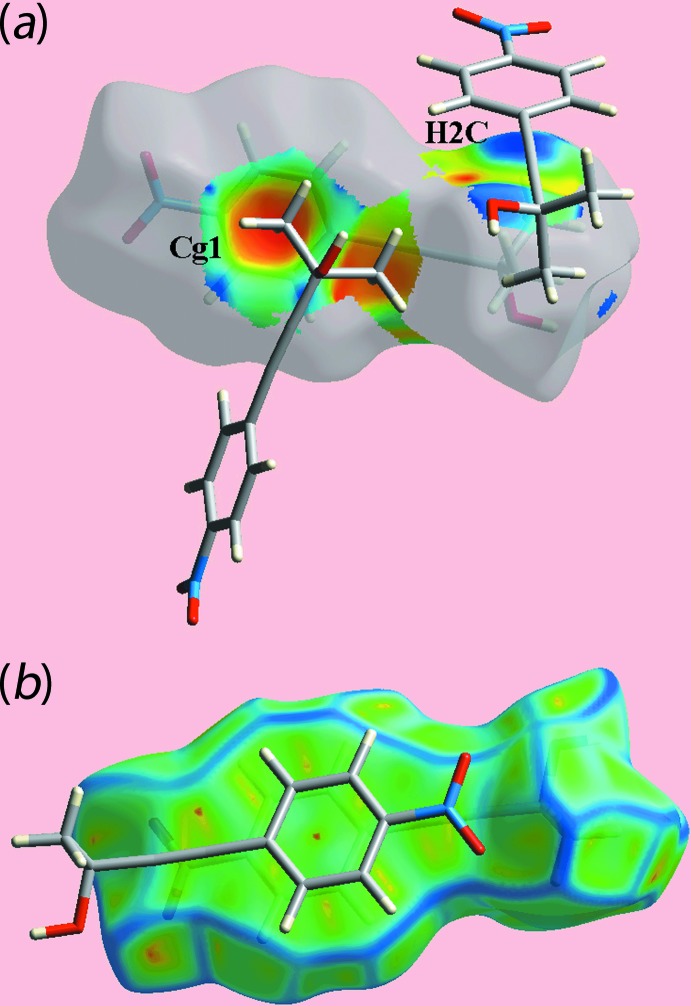
(*a*) A view of the Hirshfeld surface for (I) mapped with the shape-index property, highlighting inter­molecular C—H⋯π/π⋯H—C contacts by blue bumps and bright-orange concave regions, respectively, and (*b*) a view of the Hirshfeld surface mapped over curvedness, highlighting π—π contacts between symmetry-related (C6-C11) rings.

The Hirshfeld surface calculations for (I) were performed in accord with protocols described in a recently published paper (Tan *et al.*, 2019 ▸) employing *Crystal Explorer 17* (Turner *et al.*, 2017 ▸). On the Hirshfeld surfaces mapped over *d*_norm_ in Fig. 5 ▸(*a*), the donors and acceptors of O—H⋯O hydrogen bond involving the atoms of the hydroxyl group are characterized as bright-red spots. The faint-red spots near the phenyl-H10, H11 and nitro-O2, O3 atoms on the *d*_norm_-mapped Hirshfeld surface in Fig. 5 ▸(*b*) represent the effect of weak C—H⋯O inter­actions as listed in Table 1 ▸. The Hirshfeld surface mapped over electrostatic potential in Fig. 6 ▸ also illustrates the donors and acceptors of the indicated inter­actions through blue and red regions corresponding to positive and negative electrostatic potentials, respectively. In the view of a surface mapped with the shape-index property, Fig. 7 ▸(*a*), the C—H⋯π/π⋯H—C contacts listed in Table 2 ▸ are evident as the blue bump and a bright-orange region about the participating atoms. The overlap between benzene (C6–C11) ring of a reference mol­ecule within the Hirshfeld surface mapped over curvedness and the symmetry related ring, Fig. 7 ▸(*b*) is an indication of the π–π stacking inter­action between them [centroid–centroid distance = 3.7873 (14) Å; symmetry operation: 1 − *x*, 1 − *y*, 1 − *z*].

**Figure 8:**
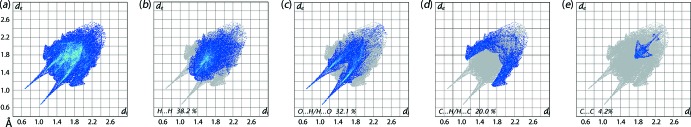
(*a*) The full two-dimensional fingerprint plot for (I) and (*b*)–(*e*) those delineated into H⋯H, O⋯H/H⋯O, C⋯H/H⋯C and C⋯C contacts, respectively.

The overall two-dimensional fingerprint plot for (I), Fig. 8 ▸(*a*), and those delineated into H⋯H, O⋯H/H⋯O, C⋯H/H⋯C and C⋯C contacts (McKinnon *et al.*, 2007 ▸) are illustrated in Fig. 8 ▸(*b*)–(*e*), respectively, and provide more information on the influence of short inter­atomic contacts upon the mol­ecular packing. The percentage contributions from the different inter­atomic contacts to the Hirshfeld surface are summarized in Table 3 ▸. The greatest contribution to the Hirshfeld surface of 38.2% are derived from H⋯H contacts but these exert a negligible influence on the packing, at least in terms of directional inter­actions, as the inter­atomic distances are greater than sum of their van der Waals radii. The pair of long spikes with their tips at *d*_e_ + *d*_i_ ∼1.8 Å in the fingerprint plot delineated into O⋯H/H⋯O contacts, Fig. 8 ▸(*c*), are due to the presence of the O—H⋯O hydrogen bond, whereas the points corresponding to comparatively weak inter­molecular C—H⋯O inter­actions, Table 1 ▸, and the short inter­atomic O⋯H/H⋯O contacts are merged within the plot, Table 2 ▸. The presence of the C—H⋯π contact, formed by the methyl-H2*C* atom and the benzene (C6–C11) ring, results in short inter­atomic C⋯H/H⋯C contacts, Table 2 ▸ and Fig. 7 ▸(*a*), and by the pair of forceps-like tips at *d*_e_ + *d*_i_ ∼2.8 Å in Fig. 8 ▸(*d*). The points corresponding to other such short inter­atomic contacts involving the acetyl­ene-C5 and methyl-C3—H3c atoms at longer separations are merged within the plot. The arrow-shaped distribution of points around *d*_e_ + *d*_i_ ∼3.6 Å in the fingerprint plot delineated into C⋯C contacts, Fig. 8 ▸(*e*), indicate π–π overlap between symmetry-related benzene (C6–C11) rings, as illustrated in Fig. 7 ▸(*b*). The small percentage contributions from the other inter­atomic contacts listed in Table 3 ▸ have negligible influence upon the mol­ecular packing as their separations are greater than the sum of the respective van der Waals radii.

## Inter­action energies   

The pairwise inter­action energies between the mol­ecules within the crystal were calculated by summing up four energy components comprising electrostatic (*E*_ele_), polarization (*E*_pol_), dispersion (*E*_dis_) and exchange-repulsion (*E*_rep_) terms after applying relevant scale factors (Turner *et al.*, 2017 ▸). These energies were obtained by using the wave function calculated at the B3LYP/6-31G(d,p) level. The strength and the nature of inter­molecular inter­actions in terms of their energies are qu­anti­tatively summarized in Table 4 ▸. The energies calculated for the different inter­molecular inter­actions indicate that the electrostatic contribution is dominant in the O—H⋯O hydrogen bond whereas the dispersive component has a significant influence due to the presence of short inter­atomic C⋯H/H⋯C and O⋯H/H⋯O contacts occurring between the same pair of mol­ecules. The C—H⋯O2(nitro) inter­action has almost the same contributions from the electrostatic and dispersive components. This is in contrast to a major contribution only from the dispersive component for the analogous contact involving the nitro-O3 atom. The dispersion energy component makes the major contribution to the relevant pairs of mol­ecules involved in other short inter­atomic contacts, Table 4 ▸, as well as in C—H⋯π and π–π stacking inter­actions. It is also evident from a comparison of the total energies of inter­molecular inter­actions, Table 4 ▸, that the O—H⋯O hydrogen bond and π–π stacking inter­action are stronger than the other inter­actions, and, of these, the inter­molecular C—H⋯O contacts are weaker than the C—H⋯π inter­actions.

**Figure 9:**
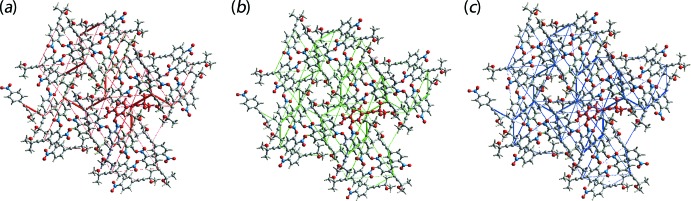
A comparison of the energy frameworks calculated for (I) and viewed down the *c* axis showing (*a*) electrostatic potential force, (*b*) dispersion force and (*c*) total energy. The energy frameworks were adjusted to the same scale factor of 30 with a cut-off value of 3 kJ mol^−1^ within 2 × 2 × 2 unit cells.

The magnitudes of inter­molecular energies are represented graphically by energy frameworks to view the supra­molecular architecture of the crystal through the cylinders joining centroids of mol­ecular pairs by using red, green and blue colour codes for the components *E*_ele_, *E*_disp_ and *E*_tot_, respectively, Fig. 9 ▸. The radius of the cylinder is proportional to the magnitude of inter­action energy, which are adjusted to the same scale factor of 30 with a cut-off value of 3 kJ mol^−1^ within 2 × 2 × 2 unit cells.

## Non-covalent inter­action plots   

**Figure 10:**
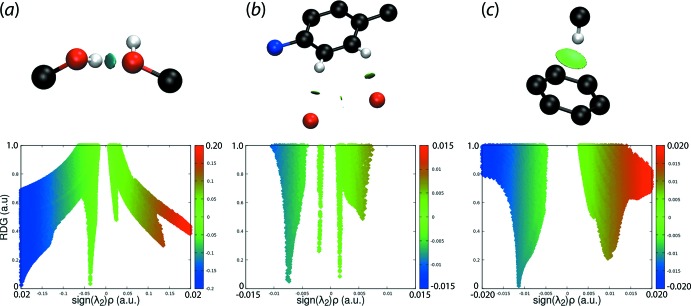
Non-covalent inter­action plots for (*a*) hy­droxy-O—H⋯O(hy­droxy) hydrogen bonding, (*b*) the phenyl-C—H⋯O(nitro) inter­actions and (*c*) the methyl-C—H⋯π(C11–C16) inter­actions.

Non-covalent inter­action plot (NCIplot) analyses provide a visual representation of the nature of the contact between specified species in crystals (Johnson *et al.*, 2010 ▸; Contreras-Garcá *et al.*, 2011 ▸). This method is based on the electron density (and derivatives) and was employed in the present study to confirm the nature of some of the specified inter­molecular contacts. The colour-based isosurfaces generated correspond to the values of sign(λ^2^)*ρ*(r), where *ρ* is the electron density and λ^2^ is the second eigenvalue of the Hessian matrix of *ρ*. Crucially, through a three-colour scheme, a specific inter­action can be identified as being attractive or otherwise. Thus, a green isosurface indicates a weakly attractive inter­action whereas a blue isosurface indicates an attractive inter­action; a repulsive inter­action appears red. The isosurfaces for three identified inter­molecular inter­actions are given in the upper view of Fig. 10 ▸. Thus, in Fig. 10 ▸(*a*), a green isosurface is apparent for the conventional hy­droxy-O—H⋯O(hy­droxy) hydrogen bond. Similarly, green isosurfaces are seen between the inter­acting atoms involved in the phenyl-C—H⋯O(nitro), Fig. 10 ▸(*b*), and the methyl-C—H⋯π(C11–C16), Fig. 10 ▸(*c*), inter­actions.

The lower views of Fig. 10 ▸, show the plots of the RDG *versus* sign(λ^2^)*ρ*(r). The non-covalent inter­action peaks appear at density values less than 0.0 atomic units, consistent with their being weakly attractive inter­actions.

## Database survey   

**Figure 11:**
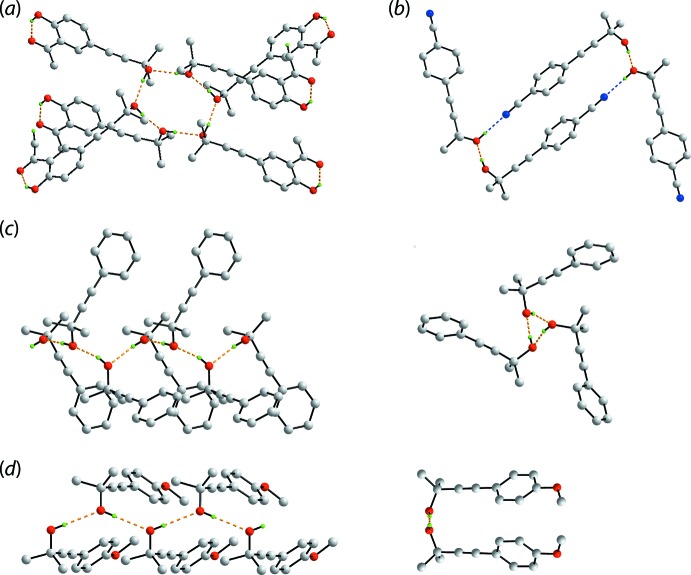
Supra­molecular association *via* hy­droxy-O—H⋯O(hy­droxy) hydrogen bonds in (II)–(IV): (*a*) hexa­meric cluster in (V), (*b*) dimeric aggregate sustained by additional hy­droxy-O—H⋯N(cyano) hydrogen bonds in (III), (*c*) views of the supra­molecular chain in (II) with non-crystallographic threefold symmetry and (*d*) views of the zigzag supra­molecular chain in (IV).

There are four literature precedents for (I) with varying substitution patterns in the appended benzene ring. These are the unsubstituted ‘parent’ compound [(II); FESMEV; Singelenberg & van Eijck, 1987 ▸], and the 4-cyano [(III}; HEFDAA; Clegg, 2017 ▸], 4-meth­oxy [(IV); YUQPEG; Eissmann *et al.*, 2010 ▸] and 3-acetyl-4-hy­droxy [(V); UVETAS; Hübscher *et al.*, 2016 ▸] derivatives. Selected geometric parameters for (I)–(IV) are collated in Table 5 ▸. Of particular inter­est in the mode of supra­molecular association in their crystals. As seen from Fig. 11 ▸, four distinct patterns appear. In (V), three independent mol­ecules comprise the asymmetric unit and these associate about a centre of inversion in space group *P*2_1_/*c* to form a hexa­meric clusters *via* hy­droxy-O—H⋯O(hy­droxy) hydrogen bonds as seen in (I), Fig. 11 ▸(*a*); intra­molecular hy­droxy-O—H⋯O(carbon­yl) hydrogen bonds are also apparent. In (III), the two independent mol­ecules comprising the asymmetric unit associate about a centre of inversion in space group *P*2_1_/*n* into a supra­molecular dimer *via* pairs of hy­droxy-O—H⋯O(hy­droxy) and hy­droxy-O—H⋯N(cyano) hydrogen bonds as shown in Fig. 11 ▸(*b*). In this case, one independent hy­droxy-oxygen atom and one cyano-nitro­gen atom do not accept a hydrogen-bonding inter­action. Three crystallographically independent mol­ecules are also found in (II) (space group *Pca*2_1_) and these self-associate to form a supra­molecular chain *via* hy­droxy-O—H⋯O(hy­droxy) hydrogen bonds with non-crystallographic threefold symmetry, Fig. 11 ▸(*c*). Finally, zigzag supra­molecular chains sustained by hy­droxy-O—H⋯O(hy­droxy) hydrogen bonds are found in the crystal of (IV), Fig. 11 ▸(*d*) in space group *Pbca*.

## Synthesis and crystallization   

The title compound was prepared as per the literature procedure (Bleicher *et al.*, 1998 ▸). Yield: 87%. Yellow solid, m.p. 377–379 K. ^1^H NMR (400 MHz, CDCl_3_): δ = 8.16 (*dt*, *J* = 8.9, 2.2 Hz, 2H), 7.54 (*dt*, *J* = 8.9, 2.2 Hz, 2H), 2.24 (*s*, 1H) and 1.63 (*s*, 6H) ppm. ^13^C NMR (101 MHz, CDCl_3_): δ = 147.2, 132.5, 129.8, 123.6, 99.2, 80.5, 66.7 and 31.3 ppm. Irregular colourless crystals of (I) for the X-ray study were grown by slow evaporation of its ethyl acetate solution.

## Refinement details   

Crystal data, data collection and structure refinement details are summarized in Table 6 ▸. The carbon-bound H atoms were placed in calculated positions (C—H = 0.93–0.96 Å) and were included in the refinement in the riding-model approximation, with *U*_iso_(H) set to 1.2–1.5*U*_eq_(C). The O-bound H atom was refined with a distance restraint of 0.82±0.01 Å, and with *U*_iso_(H) = 1.5*U*_eq_(O).

## Supplementary Material

Crystal structure: contains datablock(s) I, global. DOI: 10.1107/S2056989019010284/hb7841sup1.cif

Structure factors: contains datablock(s) I. DOI: 10.1107/S2056989019010284/hb7841Isup2.hkl

CCDC reference: 1941466

Additional supporting information:  crystallographic information; 3D view; checkCIF report

## Figures and Tables

**Table 1:** Hydrogen-bond geometry (Å, °)

*D*—H⋯*A*	*D*—H	H⋯*A*	*D*⋯*A*	*D*—H⋯*A*
O1—H1*O*⋯O1^i^	0.82	1.87	2.682 (2)	173
C10—H10⋯O3^ii^	0.93	2.67	3.548 (3)	157
C11—H11⋯O2^iii^	0.93	2.68	3.467 (3)	143

Symmetry codes: (i) 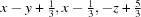; (ii) 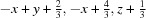; (iii) 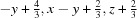.

**Table 2:** Summary of short inter­atomic contacts (Å) in (I) The inter­atomic distances are calculated in *Crystal Explorer 17* (Turner *et al.*, 2017 ▸) whereby the *X*—H bond lengths are adjusted to their neutron values.

Contact	Distance	Symmetry operation
O1⋯H3*A*	2.71	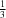 + *y*, 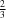 − *x* + *y*, 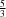 − *z*
O2⋯H2*B*	2.69	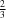 − *y*, 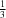 + *x* − *y*, − 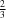 + *z*
O3⋯H2*A*	2.69	1 − *x*, 1 − *y*, 1 − *z*
C1⋯H1*O*	2.85	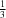 + *y*, 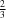 − *x* + *y*, 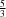 − *z*
C5⋯H3*C*	2.79	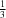 + *y*, 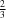 − *x* + *y*, 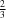 − *z*
C7⋯H2*C*	2.85	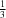 + *y*, 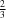 − *x* + *y*, 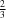 − *z*
C8⋯H2*C*	2.80	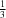 + *y*, 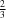 − *x* + *y*, 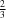 − *z*

**Table 3:** Percentage contributions of inter­atomic contacts to the Hirshfeld surface for (I)

Contact	Percentage contribution
H⋯H	38.2
O⋯H/H⋯O	32.1
C⋯H/H⋯C	20.0
C⋯C	4.2
N⋯O/O⋯N	1.7
O⋯O	1.6
C⋯N/N⋯C	1.0
N⋯H/H⋯N	0.8
C⋯O/O⋯C	0.4

**Table 4:** Summary of inter­action energies (kJ mol^−1^) calculated for (I)

Contact	*R* (Å)	*E*_ele_	*E*_pol_	*E*_dis_	*E*_rep_	*E*_tot_
O1—H1*O*⋯O1^i^						
H3*A*⋯O1^i^	8.80	−52.3	−12.0	−18.8	72.7	−35.7
H1*O*⋯C1^i^						
C10—H10⋯O3^ii^	8.28	−3.7	−1.4	−9.2	4.9	−9.8
C11—H11⋯O2^iii^	9.51	−5.8	−1.7	−5.7	5.0	−9.6
O3⋯H2*A* ^iv^						
(C6–C11)⋯(C6–C11)^iv^	4.25	−9.4	−1.8	−47.1	28.9	−34.4
H3*C*⋯C5^v^						
H2*C*⋯C7^v^						
H2*C*⋯C8^v^	5.78	−2.1	−0.7	−28.6	18.2	−16.4
C2—H2*C*⋯(C6–C11)^v^						

Symmetry codes: (i) 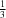 + *x* − *y*, 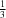 − *x*, 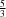 − *z*; (ii) 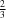 − *x* + *y*, 4/3 − *x*, 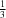 + *z*; (iii) 4/3 − *y*, 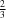 + *x* − *y*, 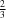 + *z*; (iv) 1 − *x*, 1 − *y*, 1 − *z*; (v) 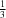 + *x* − *y*, − 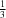 + *x*, 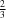 − *z*.

**Table 5:** Geometric data (Å, °) for related 2-methyl-4-(ar­yl)but-3-yn-2-ol mol­ecules

Compound	*Z*′	C_ring_—C_acetyl­ene_	C_acetyl­ene_—C_acetyl­ene_	C_acetyl­ene_—C_quaternary_	Supra­molecular motif	Reference
(I)	1	1.438 (3)	1.189 (3)	1.471 (3)	hexa­mer	This work
(II)	3	1.443 (5)	1.211 (5)	1.454 (5)	chain	Singelenberg & van Eijck (1987 ▸)
		1.437 (6)	1.192 (6)	1.479 (6)		
		1.437 (5)	1.189 (5)	1.479 (5)		
(III)	2	1.441 (2)	1.193 (2)	1.490 (2)	dimer	Clegg (2017 ▸)
		1.435 (2)	1.1895 (2)	1.480 (2)		
(IV)	1	1.4377 (16)	1.2000 (16)	1.4791 (16)	chain	Eissmann *et al.* (2010 ▸)
(V)	3	1.4418 (18)	1.1951 (19)	1.4764 (19)	hexa­mer	Hübscher *et al.* (2016 ▸)
		1.444 (2)	1.194 (2)	1.4859 (19)		
		1.4402 (19)	1.1904 (19)	1.4723 (18)		

**Table 6:** Experimental details

Crystal data	
Chemical formula	C_11_H_11_NO_3_
*M*_r_	205.21
Crystal system, space group	Trigonal, *R* 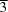 :*H*
Temperature (K)	296
*a*, *c* (Å)	26.3146 (14), 8.1205 (5)
*V* (Å^3^)	4869.8 (6)
*Z*	18
Radiation type	Mo *K*α
μ (mm^−1^)	0.09
Crystal size (mm)	0.34 × 0.28 × 0.16

Data collection	
Diffractometer	Bruker APEXII CCD
Absorption correction	Multi-scan (*SADABS*; Sheldrick, 1996 ▸)
*T*_min_, *T*_max_	0.440, 0.745
No. of measured, independent and observed [*I* > 2σ(*I*)] reflections	10643, 2230, 1513
*R*_int_	0.080
(sin θ/λ)_max_ (Å^−1^)	0.627

Refinement	
*R*[*F* ^2^ > 2σ(*F* ^2^)], *wR*(*F* ^2^), *S*	0.053, 0.149, 1.05
No. of reflections	2230
No. of parameters	139
No. of restraints	1
H-atom treatment	H-atom parameters constrained
Δρ_max_, Δρ_min_ (e Å^−3^)	0.16, −0.27

Computer programs: *APEX2* and *SAINT* (Bruker, 2009 ▸), *SIR2014* (Burla *et al.*, 2015 ▸), *SHELXL2018/3* (Sheldrick, 2015 ▸), *ORTEP-3 for Windows* (Farrugia, 2012 ▸), *DIAMOND* (Brandenburg, 2006 ▸), *MarvinSketch* (ChemAxon, 2010 ▸) and *publCIF* (Westrip, 2010 ▸).

## supplementary crystallographic information

### Crystal data

**Table:**

C_11_H_11_NO_3_	*D*_x_ = 1.260 Mg m^−^^3^
*M**_r_* = 205.21	Mo *K*α radiation, λ = 0.71073 Å
Trigonal, *R*3:*H*	Cell parameters from 2006 reflections
*a* = 26.3146 (14) Å	θ = 2.7–23.9°
*c* = 8.1205 (5) Å	µ = 0.09 mm^−^^1^
*V* = 4869.8 (6) Å^3^	*T* = 296 K
*Z* = 18	Irregular, colourles
*F*(000) = 1944	0.34 × 0.28 × 0.16 mm

### Data collection

**Table:**

Bruker APEXII CCD diffractometer	1513 reflections with *I* > 2σ(*I*)
φ and ω scans	*R*_int_ = 0.080
Absorption correction: multi-scan (SADABS; Sheldrick, 1996)	θ_max_ = 26.4°, θ_min_ = 1.6°
*T*_min_ = 0.440, *T*_max_ = 0.745	*h* = −32→32
10643 measured reflections	*k* = −32→32
2230 independent reflections	*l* = −9→10

### Refinement

**Table:**

Refinement on *F*^2^	Primary atom site location: structure-invariant direct methods
Least-squares matrix: full	Secondary atom site location: difference Fourier map
*R*[*F*^2^ > 2σ(*F*^2^)] = 0.053	Hydrogen site location: inferred from neighbouring sites
*wR*(*F*^2^) = 0.149	H-atom parameters constrained
*S* = 1.05	*w* = 1/[σ^2^(*F*_o_^2^) + (0.0511*P*)^2^ + 3.9317*P*] where *P* = (*F*_o_^2^ + 2*F*_c_^2^)/3
2230 reflections	(Δ/σ)_max_ < 0.001
139 parameters	Δρ_max_ = 0.16 e Å^−^^3^
1 restraint	Δρ_min_ = −0.27 e Å^−^^3^

### Special details

**Table:**

Geometry. All esds (except the esd in the dihedral angle between two l.s. planes) are estimated using the full covariance matrix. The cell esds are taken into account individually in the estimation of esds in distances, angles and torsion angles; correlations between esds in cell parameters are only used when they are defined by crystal symmetry. An approximate (isotropic) treatment of cell esds is used for estimating esds involving l.s. planes.

### Fractional atomic coordinates and isotropic or equivalent isotropic displacement parameters (Å^2^)

**Table:**

	*x*	*y*	*z*	*U*_iso_*/*U*_eq_	
O1	0.56993 (7)	0.33529 (6)	0.78650 (17)	0.0513 (4)	
H1O	0.569241	0.305973	0.823659	0.077*	
O2	0.54778 (9)	0.61319 (8)	0.0578 (2)	0.0811 (6)	
O3	0.62154 (8)	0.66736 (8)	0.2126 (3)	0.0785 (6)	
N1	0.58062 (9)	0.61978 (9)	0.1723 (3)	0.0565 (5)	
C1	0.53437 (9)	0.31996 (8)	0.6425 (2)	0.0388 (5)	
C2	0.47123 (10)	0.27726 (10)	0.6889 (3)	0.0612 (7)	
H2A	0.459150	0.293892	0.775102	0.092*	
H2B	0.468058	0.241200	0.726620	0.092*	
H2C	0.446510	0.269637	0.594458	0.092*	
C3	0.55665 (12)	0.29361 (11)	0.5140 (3)	0.0634 (7)	
H3A	0.551638	0.257044	0.553954	0.095*	
H3B	0.597504	0.320235	0.493279	0.095*	
H3C	0.534860	0.286951	0.413769	0.095*	
C4	0.54001 (9)	0.37468 (9)	0.5772 (2)	0.0439 (5)	
C5	0.54464 (10)	0.41762 (9)	0.5145 (2)	0.0458 (5)	
C6	0.55281 (9)	0.46950 (9)	0.4317 (2)	0.0407 (5)	
C7	0.51138 (9)	0.46599 (9)	0.3192 (2)	0.0424 (5)	
H7	0.477559	0.430184	0.300298	0.051*	
C8	0.52018 (9)	0.51543 (9)	0.2351 (2)	0.0441 (5)	
H8	0.492523	0.513284	0.159933	0.053*	
C9	0.57045 (9)	0.56768 (9)	0.2648 (2)	0.0416 (5)	
C10	0.61185 (10)	0.57274 (9)	0.3773 (3)	0.0515 (6)	
H10	0.645262	0.608821	0.396842	0.062*	
C11	0.60276 (10)	0.52332 (10)	0.4602 (3)	0.0509 (6)	
H11	0.630422	0.526000	0.536242	0.061*	

### Atomic displacement parameters (Å^2^)

**Table:**

	*U*^11^	*U*^22^	*U*^33^	*U*^12^	*U*^13^	*U*^23^
O1	0.0636 (10)	0.0378 (8)	0.0474 (8)	0.0216 (7)	−0.0238 (7)	−0.0036 (6)
O2	0.0870 (14)	0.0812 (13)	0.0791 (12)	0.0450 (11)	−0.0075 (11)	0.0315 (10)
O3	0.0734 (13)	0.0474 (10)	0.1082 (15)	0.0254 (10)	0.0056 (11)	0.0178 (10)
N1	0.0592 (12)	0.0508 (12)	0.0661 (13)	0.0324 (11)	0.0118 (10)	0.0164 (9)
C1	0.0463 (11)	0.0376 (10)	0.0322 (9)	0.0206 (9)	−0.0083 (8)	−0.0026 (8)
C2	0.0501 (14)	0.0552 (14)	0.0690 (15)	0.0191 (12)	−0.0049 (11)	0.0064 (11)
C3	0.0898 (19)	0.0674 (16)	0.0493 (13)	0.0514 (15)	−0.0007 (12)	−0.0056 (11)
C4	0.0518 (12)	0.0460 (12)	0.0365 (10)	0.0264 (10)	−0.0029 (9)	0.0014 (9)
C5	0.0577 (13)	0.0484 (12)	0.0364 (10)	0.0303 (11)	0.0001 (9)	0.0009 (9)
C6	0.0535 (12)	0.0444 (11)	0.0311 (9)	0.0297 (10)	0.0052 (8)	0.0026 (8)
C7	0.0452 (11)	0.0423 (11)	0.0400 (10)	0.0223 (10)	0.0024 (9)	0.0009 (8)
C8	0.0480 (12)	0.0543 (13)	0.0379 (10)	0.0317 (11)	0.0011 (9)	0.0045 (9)
C9	0.0486 (12)	0.0431 (11)	0.0413 (10)	0.0291 (10)	0.0088 (9)	0.0080 (8)
C10	0.0491 (13)	0.0422 (12)	0.0598 (13)	0.0203 (10)	−0.0055 (10)	−0.0006 (10)
C11	0.0566 (14)	0.0538 (13)	0.0468 (11)	0.0310 (11)	−0.0109 (10)	−0.0005 (10)

### Geometric parameters (Å, º)

**Table:**

O1—C1	1.424 (2)	C3—H3C	0.9600
O1—H1O	0.8200	C4—C5	1.189 (3)
O2—N1	1.221 (3)	C5—C6	1.438 (3)
O3—N1	1.219 (2)	C6—C11	1.387 (3)
N1—C9	1.466 (3)	C6—C7	1.390 (3)
C1—C4	1.471 (3)	C7—C8	1.382 (3)
C1—C2	1.516 (3)	C7—H7	0.9300
C1—C3	1.523 (3)	C8—C9	1.371 (3)
C2—H2A	0.9600	C8—H8	0.9300
C2—H2B	0.9600	C9—C10	1.376 (3)
C2—H2C	0.9600	C10—C11	1.375 (3)
C3—H3A	0.9600	C10—H10	0.9300
C3—H3B	0.9600	C11—H11	0.9300
			
C1—O1—H1O	109.5	H3B—C3—H3C	109.5
O3—N1—O2	123.3 (2)	C5—C4—C1	175.7 (2)
O3—N1—C9	118.5 (2)	C4—C5—C6	176.5 (2)
O2—N1—C9	118.2 (2)	C11—C6—C7	119.25 (18)
O1—C1—C4	106.76 (15)	C11—C6—C5	120.46 (18)
O1—C1—C2	109.11 (16)	C7—C6—C5	120.27 (19)
C4—C1—C2	110.61 (18)	C8—C7—C6	120.36 (19)
O1—C1—C3	110.10 (17)	C8—C7—H7	119.8
C4—C1—C3	108.98 (16)	C6—C7—H7	119.8
C2—C1—C3	111.20 (18)	C9—C8—C7	118.75 (18)
C1—C2—H2A	109.5	C9—C8—H8	120.6
C1—C2—H2B	109.5	C7—C8—H8	120.6
H2A—C2—H2B	109.5	C8—C9—C10	122.25 (18)
C1—C2—H2C	109.5	C8—C9—N1	118.80 (18)
H2A—C2—H2C	109.5	C10—C9—N1	118.95 (19)
H2B—C2—H2C	109.5	C11—C10—C9	118.6 (2)
C1—C3—H3A	109.5	C11—C10—H10	120.7
C1—C3—H3B	109.5	C9—C10—H10	120.7
H3A—C3—H3B	109.5	C10—C11—C6	120.79 (19)
C1—C3—H3C	109.5	C10—C11—H11	119.6
H3A—C3—H3C	109.5	C6—C11—H11	119.6
			
C11—C6—C7—C8	0.7 (3)	O3—N1—C9—C10	−9.2 (3)
C5—C6—C7—C8	−177.66 (17)	O2—N1—C9—C10	170.3 (2)
C6—C7—C8—C9	0.2 (3)	C8—C9—C10—C11	1.2 (3)
C7—C8—C9—C10	−1.2 (3)	N1—C9—C10—C11	−178.21 (19)
C7—C8—C9—N1	178.28 (17)	C9—C10—C11—C6	−0.3 (3)
O3—N1—C9—C8	171.3 (2)	C7—C6—C11—C10	−0.7 (3)
O2—N1—C9—C8	−9.2 (3)	C5—C6—C11—C10	177.72 (19)

### Hydrogen-bond geometry (Å, º)

**Table:**

*D*—H···*A*	*D*—H	H···*A*	*D*···*A*	*D*—H···*A*
O1—H1*O*···O1^i^	0.82	1.87	2.682 (2)	173
C10—H10···O3^ii^	0.93	2.67	3.548 (3)	157
C11—H11···O2^iii^	0.93	2.68	3.467 (3)	143

Symmetry codes: (i) *x*−*y*+1/3, *x*−1/3, −*z*+5/3; (ii) −*x*+*y*+2/3, −*x*+4/3, *z*+1/3; (iii) −*y*+4/3, *x*−*y*+2/3, *z*+2/3.
